# Investigating the Effect of Basic Amino Acids and Glucosamine on the Solubility of Ibuprofen and Piroxicam

**DOI:** 10.34172/apb.2023.067

**Published:** 2022-12-10

**Authors:** Hadi Valizadeh, Somayeh Mahdinloo, Negin Zakeri, Muhammad Sarfraz, Saeed Nezafat, Parvin Zakeri-Milani

**Affiliations:** ^1^Drug Applied Research Center and Faculty of Pharmacy, Tabriz University of Medical Sciences, Tabriz, Iran.; ^2^Student Research Committee, Faculty of Pharmacy, Tabriz University of Medical Sciences, Tabriz, Iran.; ^3^College of Pharmacy, Al Ain University, Al Ain 64141, United Arab Emirates.; ^4^Liver and Gastrointestinal Diseases Research Center and Faculty of Pharmacy, Tabriz University of Medical Sciences, Tabriz, Iran.

**Keywords:** Solubility, Ibuprofen, Piroxicam, Arginine, Lysine, Glucosamine

## Abstract

**Purpose:**

Poor aqueous solubility hampers the development of several compounds as pharmacological agents. Hence, preparing novel formulations with augmented absorption is a challenge in pharmaceutical industries. In this paper, we have examined the effect of basic amino acids including arginine (ARG), lysine (LYS), and glucosamine (GlucN) on the solubility of ibuprofen (IBU) and piroxicam (PXM) as drugs with limited solubility. We have also studied the effect of the dissolution media with the pH values 1.2 to 7.4.

**Methods:**

The saturation shake-flask method was used for solubility studies in the presence of amino acids. Briefly, buffer solutions containing different concentrations of amino acids were prepared. Then, an excess amount of each drug with these buffers was shaken to reach equilibrium. After 48 hours, the upper phase was separated, and solubility was calculated by reading their UV-Vis absorbance.

**Results:**

The results illustrated that amino acids increased solubility of both drugs with different ratios, which were pH and concentration-dependent. Solubility improved as the amount of amino acids went up, and this upward pattern was more robust with ARG than LYS. The presence of GlucN in citrate buffer significantly enhanced IBU solubility. The solubility of PXM in accompany of GlucN in water did not change significantly while in citrate buffer solubility enhanced specially at pH 6.

**Conclusion:**

Overall, GlucN in citrate buffer and ARG in phosphate buffer could be introduced as the most suitable media for IBU and PXM solubility improvement, respectively.

## Introduction

 Nearly 40% of the new chemicals developed from drug discoveries are water-insoluble. Poor solubility hinders dissolution and absorption leading to poor bioavailability at high doses, gastrointestinal toxicity, and failure in trial phases. Poorly water-soluble drugs are characterized as class II and class IV of the Biopharmaceutical Classification System (BCS). Formulating these kinds of drugs for oral administration is one of the scientist’s challenges.^1^

**Figure 1:**
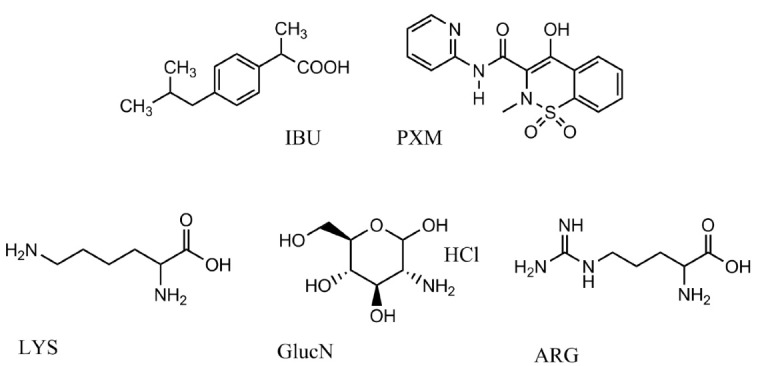
Chemical structure of ibuprofen (IBU), piroxicam (PXM), lysine (LYS), glucosamine (GlucN), and arginine (ARG)

 Ibuprofen (IBU), a chiral 2-aryl propionic acid derivative (Figure 1), is counted as a well-tolerated non-steroidal anti-inflammatory drug (NSAID) and analgesic. It is used for rheumatoid arthritis, osteoarthritis, and mild to moderate pain relief since 1969.^2,3^ Studies have revealed that 5% to 7% of hospital admissions are related to drug toxicity, and 11% to 12% of those admissions are related to non-aspirin NSAIDs. To avoid this abuse, Food and Drug Administration (FDA) and European Medicines Agency (EMA) suggested the lowest dose and shortest period of NSAID prescription.^4^

 IBU was developed as a free acid, with an approximate pK_a_ value and intrinsic water solubility of 4.4 and 0.06 mg/mL, respectively. Therefore, it will remain 99.9% unionized in the stomach. The fast emptying time of the stomach and its solubility limitation will hinder its entrance into systemic circulation. Inside the small intestine, it will be solubilized but will not permeate through the membrane since it has pH-dependent solubility and permeability.^5^

 Piroxicam (PXM) is a potent NSAID indicated for long-term and acute use in osteoarthritis and rheumatoid arthritis pain relief (Figure 1). PXM with two pK_a_ values (the first value is 1.86 and the second is 5.46) is considered a weak acid compound. It is classified as a Class II drug absorbed through the small intestine epithelium, and it takes 3 to 5 hours to reach peak plasma concentration after oral administration.

 A relatively slow absorption rate can be challenging in the clinic when a quick onset of action and pain alleviation is required.^3,6^ These shreds of evidence and the correlation between therapeutic effects and serum concentration have evoked pharmaceutical companies to develop novel formulations to augment the absorption of these drugs.

 Various techniques have been introduced to enhance the solubility and dissolution rates of class II and IV drugs. Solid dispersion,^7,8^ micronization,^9,10^ lipid-based formulations,^11,12^ solubilization in surfactant systems,^13^ using various complexing agents such as cyclodextrin,^14-16^ and use of prodrugs are some of those methods.^17,18^ However, these strategies have their limitations of being fairly expensive, having a pretty complicated formulation process, and finally troublesome quality control.^19^

 Amino acids have been extensively used to enhance the dissolution rate of drugs due to their hydrophilic properties and the ability to complex with drugs.^20^ Arginine (ARG) in Soleton^®^ complex,^21^ or ARG as a solubility increasing method for rPA (recombinant plasminogen activator),^22^ lysine (LYS) that has been used with ketoprofen in the Artrosilene^®^,^23^ and LYS ibuprofen^24^ are some formulations developed by amino acids. IBU ARG is the other example, first introduced on the market in 1994 in Spain, and now it is available in European countries as Spedifen^®^. A generic formulation of this drug has also been marketed in 2007.^25^ These amino acids have exhibited great success in the salt-forming and solubility increment of various drugs. Some of these formulations like those containing furosemide^26^ and cephalosporin have been prepared and used for more than 2 decades. Cephalosporin formulation with amino acid was claimed to be less painful in parental administration.^27^

 Basic amino acids such as LYS (pK_a_ = 10.5) and ARG (pK_a_ = 12.5) are considered quite strong bases that have precious upsides like self-buffering properties^28^ and producing less basicity in the formulation (Figure 1). They alsoproduce salts with pleasant taste that is beneficial in the formulation of oral liquids.^29^ Moreover, it is reported that ARG improves anti-inflammatory properties through nitric oxide synthesis and the production of anti-inflammatory factors indirectly. The solubility of sodium and potassium salts is affected in the gastric and intestinal sodium/potassium ions,^30^ but these amino acids are not affected by the common ion effect.

 Glucosamine (GlucN) is a naturally occurring amino monosaccharide considered the building block of cartilage in the joints (Figure 1). That is a non-toxic hydrophilic carrier, which is a highly water-soluble (320 mg/mL) compound and more stable than sulfate and N-acetyl salts of GlucN.^31,32^ It increases the solubility and permeability of poorly soluble drugs by improving particle wettability in an aqueous environment.

 Here in this article, we have tried to investigate and compare the effect of the aforementioned basic amino acids and GlucN on the solubility of IBU and PXM in media with different pH like phosphate buffer, citrate buffer, hydrochloric acid buffer, and distilled water.

## Materials and Methods

###  Materials

 Pure powder of IBU, PXM, LYS, ARG, and GlucN purchased from Zahravi Pharmaceutical Company (Iran); potassium dihydrogen phosphate (K_2_HPO_4_), sodium hydroxide (NaOH), sodium chloride (NaCl), hydrochloric acid (HCl), citric acid (C_6_H_8_O_7_), and trisodium citrate (Na_3_C_6_H_5_O_7_) were obtained from Merck (Darmstadt, Germany). Double distilled water was used during the study.

###  Buffer preparation

 In this study dissolution test has been performed in the presence of amino acids within various media, including phosphate buffer solution (pH = 7.4), hydrochloric acid buffer (pH = 1.2), citrate buffer (pH = 6 and pH = 4.2), and distilled water (pH = 6.5). Briefly, for each buffer, a suitable amount of components was transferred to a 250 mL volumetric flask and then diluted with distilled water to the volume line. Finally, the pH of the solution was examined and set using a pH meter (Germany, Metrohm SWISS mode).

###  Solubility measurements of IBU and PXM

 The saturation shake-flask method has been used for solubility studies. In brief, buffer solutions containing different concentrations of each amino acid were prepared. An excess amount of each drug was weighed in capped vials, then buffers alone and mixed with amino acids were added to the vials and the pH change was set again. After that, capped vials were shaken for about 48 hours on a shaker incubator (Korea, HYSC) at speed of 150 RPM and 37^ᵒ^C to reach equilibrium and result a saturated solution. After 48 hours the upper phase was separated and centrifuged. Filtered samples were diluted with buffer before reading their UV-Vis absorbance. Then the amount of solubility was calculated using a calibration curve considering the dilution coefficient. Each of the experiments was repeated three times.

## Results and Discussion

###  Solubility studies

 Equilibrium solubility was performed in phosphate and citrate buffer, distilled water, and hydrochloric acid buffer with pH 7.4 to 1.2, simulating a wide range of physiologically relevant pH: aqueous, physiological fluids, and gastric fluid. The results are summarized in Table 1 concerning those results for IBU, solubility in phosphate buffer (10.45 mg/mL) was greater than that in hydrochloric acid buffer (0.8 mg/mL). This marked difference can be ascribed to pH-dependent solubility, based on the pK_a_ value (4.4); IBU is a weak acid with weak ionization and poor solubility at pH 1.2. These results are in good correlation with previous studies on ketoprofen, a derivative of propionic acid used to alleviate rheumatic pain.^23^ It is reported that this acidic drug is ionized at higher pH values and demonstrates upper solubility like IBU.^8^

**Table 1:** Drugs solubility in different media

	**Medium**	**pH before incubation**	**pH after 48 hours incubation**	**Measured C**_ss_** (mg/mL)**			**C**_ss_** mean** **(mg/mL)**	**SD**
				**Sample 1**	**Sample 2**	**Sample 3**		
IBU	Phosphate buffer	7.4	7.06	10.51	10.33	10.51	10.45	0.10
	Water	6.5	6.37	3.21	3.11	3.06	3.12	0.07
	Citrate buffer	6	6.00	5.33	5.05	5.24	5.20	0.14
	hydrochloric acid buffer	1.2	1.26	0.79	0.82	0.79	0.80	0.01
PXM	Phosphate buffer	7.4	7.61	1.25	1.32	1.32	1.29	0.04
	Citrate buffer	4.2	4.68	0.28	0.23	0.25	0.25	0.02
	Citrate buffer	6	6.20	0.14	0.13	0.15	0.14	0.01

 As it was expected, the solubility results of PXM resembled IBU outcomes, and phosphate buffer showed higher solubility (1.29 mg/mL) for PXM. PXM solubility in buffers with pH 1 to 10 has been measured previously, and there were no changes in solubility up to pH = 5. However, solubility was increased promptly after that. This variation has proposed the pH-dependent solubility for this drug.^33^

###  The effect of amino acids on IBU solubility in phosphate buffer and hydrochloric acid buffer

 Hydrogen bonds play an essential role in drug-amino acid interactions. Therefore, ARG bonds to IBU strongly due to its higher hydrogen donor and acceptor groups (Figure 1); it is noteworthy that agglomeration tendency reduces in this way too. Besides, ionic interactions between IBU and ARG are more effective and higher than those between IBU and LYS. The effect of an auxiliary substance like ARG on enhancing the aqueous solubility of compounds like nateglinide and efavirenz has been studied too. The interaction between the hydrophilic portion of ARG and the hydrophobic part of the nateglinide/efavirenz-cyclodextrin complex reduced the surface tension and consequently increased the aqueous solubility.^34,35^

**Figure 2:**
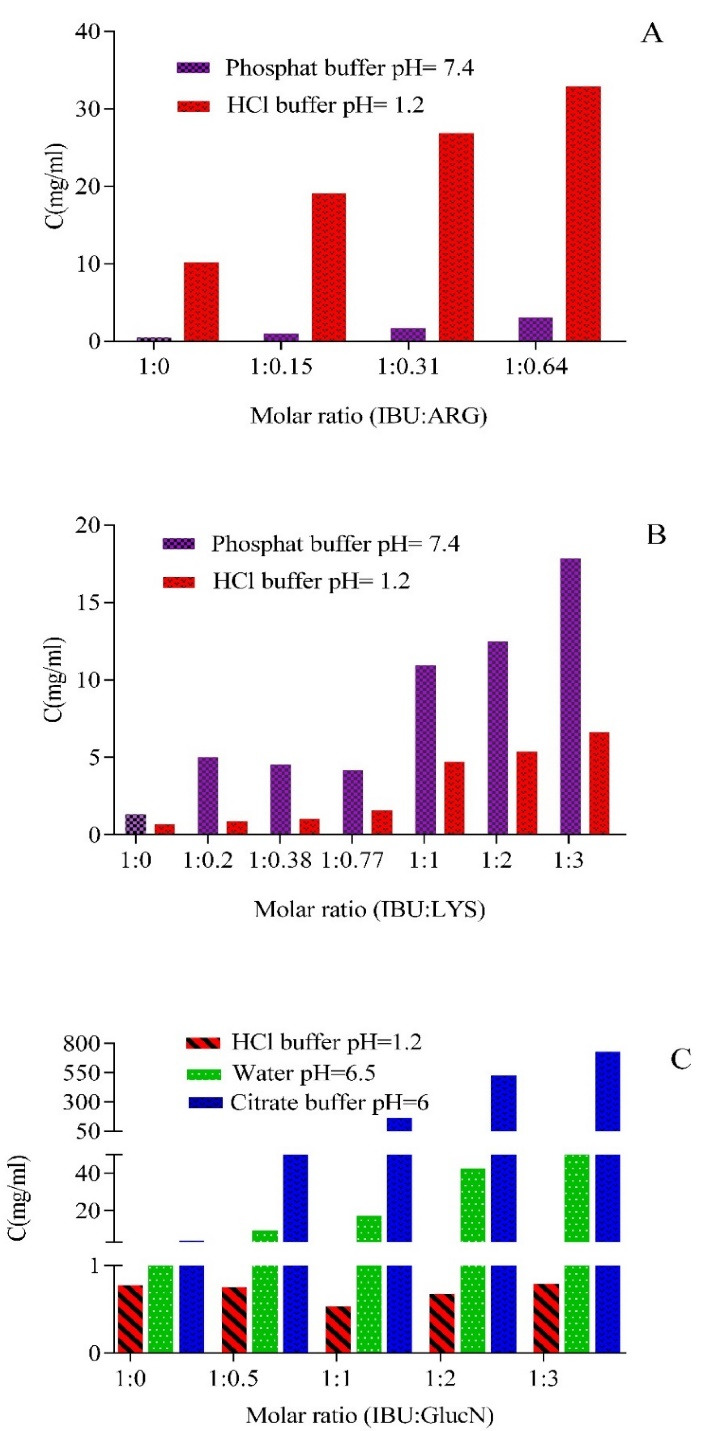
**(A)** IBU solubility in phosphate buffer and hydrochloric acid buffer supplemented with ARG. **(B)** IBU solubility in phosphate buffer and hydrochloric acid buffer supplemented with LYS. **(C)** IBU solubility in hydrochloric acid buffer, distilled water and citrate buffer supplemented with GlucN

 The solubility pattern in hydrochloric acid buffer resembles the observed trend in phosphate buffer. Solubility improved as the amount of amino acids went up, and this upward pattern was more robust with ARG than LYS (Figure 2A and 2B). GlucN was also successful in this field, but its effect was lower than LYS and ARG (Figure 2C), the reported upturn can be related to IBU interactions and amino acids. As stated before, the hydrophilic and hydrophobic interaction of IBU with ionic parts of amino acid plays an essential role in the dissolution. These interplays reduce microscopic viscosity and friction coefficient, so the mobility of particles enhances and leads to upper solubility. Furthermore, amino acid and IBU interaction decrease the interplay of water molecules and ionized amino acids, so the hydrodynamic radius of ionized particles and their movements enhance.

 A most recent study reported that pairing IBU with L-valine alkyl esters significantly enhanced IBU’s solubility in water and phosphate buffer. Also, obtained salts were slightly less soluble in buffer with pH 5.4 than in buffer with pH 7.4. Interestingly conjugates with shorter and straight alkyl chains in the L-valine ester moiety had better solubility. Lower solubility for longer hydrocarbon chains is typical which is caused by their weaker solvation; also, more tightly packed molecules need more energy to overcome the strength of intermolecular interactions.^36^

 Additionally, a previous study has examined ARG and sodium chloride’s effect on the solubility of compounds like coumarin, gluten, and octyl-gallate. Moreover, they have introduced salting-in and salting-out effects as factors that change solubility. The binding ability of ARG to the surface of proteins and aromatic groups of compounds through electron-cation interaction has been mentioned as an effective parameter.^37^ These actions may enhance the solubility of poorly soluble substances.

 In the study mentioned above, the effect of sodium chloride on solubility is also investigated, and it is stated that sodium chloride demonstrates its expected weak salting-out effect on proteins,^38^ so by increasing its concentration, solubility went down. The other fact which affects the dissolution process is the pH of the environment, which manipulates the ionization of the weak acidic compounds. Therefore, the hydrochloric acid/sodium chloride buffer reduced solubility by salting-out effect and reduction of ionization. It should be noted that amino acids represent their effect in hydrochloric acid buffer, although it is not comparable with the results of other environments.

 Some commercial products like Soleton^®^ have proved ARG’s effectiveness on dissolution rate. In this complex, cyclodextrin and ARG had been used with IBU, which led to increased solubility and bioavailability through decreasing surface tension.^21^

 Caldolor^®^ and Neoprofen are two other formulations of IBU with amino acids. Caldolor^®^ is the injectable commercial form of IBU in which ARG has been used to improve stability at the ratio of 1:0.09. This drug is the only intravenous NSAID used to alleviate fever in the USA.^39^

 Neoprofen is the LYS salt of IBU used in PDA (patent ductus arteriosus) disease. Injectable indomethacin was the preferred choice in PDA, but the FDA recommended Neoprofen for PDA infants under 32 weeks, weighing 500 to 1500 g. It is also reported that the LYS salt of IBU was more effective than indomethacin in PDA patients with fever.^40^

###  The effect of amino acids on PXM solubility in phosphate buffer and citrate buffer (pH = 4.2)

**Figure 3:**
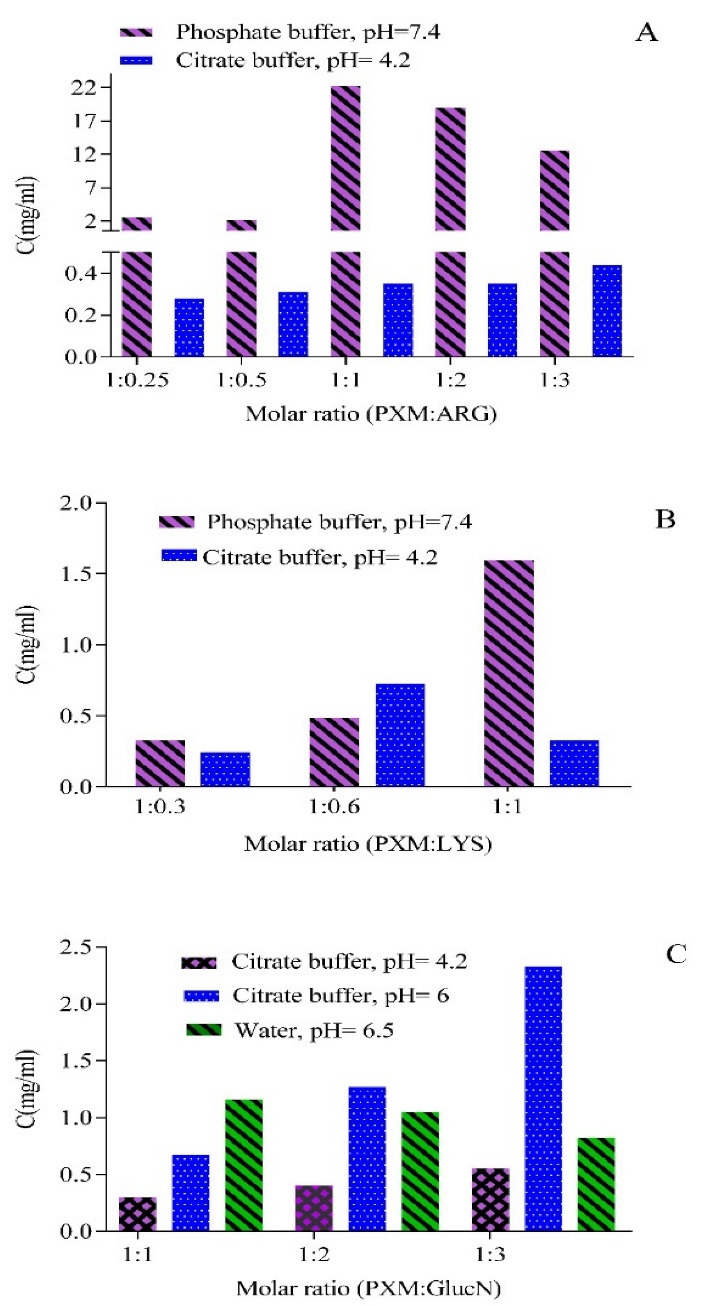
**(A)** PXM solubility in phosphate buffer and citrate buffer supplemented with ARG. **(B)** PXM solubility in phosphate buffer and citrate buffer supplemented with LYS. **(C)** PXM solubility in citrate buffer and distilled water supplemented with GlucN

 Although PXM solubility in phosphate buffer (Figure 3A) is much higher than in citrate buffer, at a concentration more than the molar ratio, 1:1 of PXM/ARG solubility goes down.

 In literature, ARG has been introduced as a solubility enhancer. It also decreases drug agglomeration by raising the number of intramolecular bonds with the drug.^41^ However, as the molar ratio gets higher than 1, the number of ionic bonds increases dramatically, and the sedimentation prevails to solubility.^42^

 The solubility increment pattern in the presence of LYS in phosphate buffer is similar to ARG, although it is a weaker pattern; because the ability of LYS in ionic bond formation is lower than ARG. Therefore, a slight enhancement is expected in LYS’s presence compared to ARG.^8^

 The hindrance of aggregation by ARG and LYS on myosin has been investigated previously; in this context, the higher myosin solubility resulted from lower aggregation and preferential interaction with acidic and aromatic amino acid residues.^43^

 PXM solubility in citrate buffer (pH = 4.2) supplemented with LYS did not follow a specific trend (Figure 3B). Previous studies have illustrated that LYS degradation follows a zero-order kinetic, so pH and temperature are the two significant stability factors. The stability of LYS in lower degree and higher pH values are improved, and it shows better efficacy on solubility. It seems degradation is the reason for that fluctuation in the solubility pattern.^44^

###  The effect of GlucN on IBU and PXM solubility in citrate buffer (pH = 6) and distilled water

 According to the summary of IBU dissolution in the presence of GlucN in citrate buffer (pH = 6), which is reported in Figure 2C, by increasing the concentration of GlucN, pH decreased, but solubility increased. This upward pattern is stronger than the reported trend for LYS and ARG.

 IBU solubility in distilled water with a pH equal to 6.5 in the presence of GlucN follows an upward pattern. However, this increase in water was lower than the amount reported in citrate buffer.^8^

 The study on PXM supports the solubility increment with GlucN. This drug illustrates weak water solubility like IBU, using a hydrophilic carrier enhances its solubility and dissolution rate. A research illustrated that formulation efficiency improves as the hydrophilic carrier rises since PXM is more susceptible to agglomeration than GlucN.^45^ Additionally, dissolution data of the physical mixture of Ketoprofen showed that the drug mixture with hydrophilic carriers like GlucN resulted in more significant wetting and solubility than pure Ketoprofen.^46^ Therefore, GlucN increment in a formulation could be a beneficial factor to improve solubility.

 PXM solubility in GlucN in water does not change significantly, but for citrate buffer (pH = 4.2) the solubility of the drug enhanced with amino acid addition, furthermore, enhancing the pH to 6 improved the solubility. These results were in accordance with previous studies. Researchers that have conducted FTIR spectra, reported hydrogen bond formation between OH groups of GlucN and sulfoxide/carbonyl groups of PXM. As mentioned before, these interactions are among the solubility enhancement factors.^47^

## Conclusion

 In this paper, the effect of amino acids on the solubility of IBU and PXM has been studied. The saturation shake-flask method and buffers with various pH ranges of 1.2-7.4 were used reminiscent: of aqueous, gastric, and physiological fluids.

 For IBU, equilibrium solubility in phosphate buffer was more excellent than that in hydrochloric acid/sodium chloride buffer. This noticeable variation could be ascribed to pH-dependent solubility since IBP is a weak acid with weak ionization and poor solubility at pH 1.2. This fact is also applicable to PXM since this drug is a weak acid too.

 The presence of amino acids enhanced the solubility of both drugs. Amino acids with hydrophilic properties act as a counter ion, improving solubility. A meaningful subject that should be noted in their application is the pH and temperature of the environment; as aforementioned, the stability of amino acids like LYS is affected by these factors. This phenomenon led to a fluctuation in PXM solubility at pH = 4.2.

 The presence of GlucN in citrate buffer (pH = 6) increased IBU solubility and decreased the pH value of the media. A similar but weaker pattern occurred in distilled water too. PXM solubility in GlucN in water did not change significantly, but citrate buffer improved solubility specially at pH 6.

 Overall, based on our evaluations, phosphate buffer was the suitable media for the dissolution of both drugs in accompany of ARG and LYS, while for GlucN, citrate buffer was the best. Also, GlucN in citrate buffer (pH = 6) and ARG within phosphate buffer (pH = 7.4) could be introduced as the best media to enhance the solubility of IBU and PXM, respectively.

## Acknowledgments

 This article is written based on Pharm.D thesis submitted by Negin Zakeri (Dissertation No.106) and Saeed Nezafat (Dissertation No.129) in the Faculty of Pharmacy, Tabriz University of Medical Sciences, Iran.

## Competing Interests

 There is no conflict of interest to declare.

## Ethical Approval

 The ethics committee approved the studies’ proposals (IR.TBZMED.REC.1396.314, IR.TBZMED.REC.1396.858).
